# Emulsion-Based Coatings for Preservation of Meat and Related Products

**DOI:** 10.3390/foods12040832

**Published:** 2023-02-15

**Authors:** Shweta Gautam, Lubomír Lapčík, Barbora Lapčíková, Robert Gál

**Affiliations:** 1 Department of Foodstuff Technology, Faculty of Technology, Tomas Bata University in Zlin, Nam. T.G. Masaryka 275, 762 72 Zlin, Czech Republic; 2 Department of Physical Chemistry, Faculty of Science, Palacky University in Olomouc, 17. Listopadu 12, 771 46 Olomouc, Czech Republic

**Keywords:** emulsion coatings, rheology, phase separation, thermal analysis, antimicrobial agents, meat

## Abstract

One of the biggest challenges faced by the meat industry is maintaining the freshness of meat while extending its shelf life. Advanced packaging systems and food preservation techniques are highly beneficial in this regard. However, the energy crisis and environmental pollution demand an economically feasible and environmentally sustainable preservation method. Emulsion coatings (ECs) are highly trending in the food packaging industry. Efficiently developed coatings can preserve food, increase nutritional composition, and control antioxidants’ release simultaneously. However, their construction has many challenges, especially for meat. Therefore, the following review focuses on the essential aspects of developing ECs for meat. The study begins by classifying emulsions based on composition and particle size, followed by a discussion on the physical properties, such as ingredient separation, rheology, and thermal characteristics. Furthermore, it discusses the lipid and protein oxidation and antimicrobial characteristics of ECs, which are necessary for other aspects to be relevant. Lastly, the review presents the limitations of the literature while discussing the future trends. ECs fabricated with antimicrobial/antioxidant properties present promising results in increasing the shelf life of meat while preserving its sensory aspects. In general, ECs are highly sustainable and effective packaging systems for meat industries.

## 1. Introduction

Fresh meat is a highly perishable food due to its complex composition and animal variety (Table 1). It attracts a wide variety of microbes and pathogens. Its freshness is impacted by the slaughtering conditions, gut microflora, storage parameters (moisture, atmospheric oxygen, light, and temperature) and internal enzymatic reactions of the animal [1]. 

Meat decay is a rapid process and begins as soon as the animal is slaughtered. The three main spoilage mechanisms are microbial, enzymatic, and lipid oxidation spoilage. The skin, intestinal tract, and the slaughtering and storage conditions of the animal are foremost sources of microbial spoilage. Spoilage bacteria most found include species of *Pseudomonas* and *Streptococcus*, among other classes, whereas the most commonly found spoilage molds are *Cladosporium* and *Sporotrichum*, among other classes. The intestinal enzymes that chemically combine with organic compounds and initiate deterioration reactions in meat cause enzymatic spoilage [6,7]. Lipid autoxidation begins when blood circulation and metabolic processes cease. 

Meat preservation aims to prevent detrimental reactions (lipid and protein oxidation and microbial decay) increase meat’s shelf-life and freshness, conserve nutrients, and most importantly, protect consumers. Furthermore, it aims to produce meat that meets and exceeds consumer expectations [8]. Multiple preservation methods have been thoroughly researched, including smoking, chilling, modified atmosphere packaging, active packaging, chemical preservatives, freezing, pickling, and edible packaging, all of which are advantageous according to the requirements of the consumer market. The increase in pollution demands environmentally sustainable methods and novel packaging systems for meat and associated products. 

Edible films (EFs) and coatings (ECs) are food-based packaging systems applied to food to safeguard it from detrimental changes and ensure preservation. The concept of EFs and ECs can be dated back to ancient China, where lipid coating was applied on lemons and oranges. Lipid coating was also used similarly in the sixteenth century in the US to preserve fruits. Since then, the idea of edible films and coatings has achieved incredible advancements. The principal difference between EFs and ECs lies in the application method. EFs are first prepared as a laminate in one or multiple layers, dried, and then applied on the food products, whereas ECs are either sprayed on the products, or the products are immersed in the EC and then dried. The major challenge in preparing the films and coatings is the selection of appropriate raw materials that will serve all the required mechanical and barrier properties and their optimum ratio. Suffice it to say that one composition cannot be generalized for a food class, creating a spacious area for the researchers to explore.

An EC is a blend of two or more immiscible liquids made miscible by adding an emulsifier. Emulsions can be classified conventionally based on their composition as O/W (oil-in-water) or W/O (water-in-oil). They can also be classified based on the number of phases as a double phase/multiple phase emulsion, wherein one emulsion is dispersed into another liquid or another emulsion, for example, O/W/O (oil-in water in-oil), or W/O/W (water-in oil in-water) emulsion. From the food packaging aspect, multiple emulsions are more advantageous than conventional ones. Multiple emulsions can be used efficiently to hold more than one functional ingredient, formulated strategically for the controlled release of certain ingredients, and they can be developed to isolate certain ingredients that might react otherwise. The food industry exploits these qualities of multiple emulsions to make complex food ECs.

The following review presents a brief classification of ECs based on the composition of the emulsions and on the particle size, followed by a comprehensive study of the three most essential physical properties (ingredient separation, rheology, and thermal stability) of consideration to prepare an efficient emulsion. Lastly, the advantages of ECs, limitations in current research, and scopes for future prospects are briefly discussed. The data collected have been limited to those from the past 12 years, i.e., 2010 to 2022, from prominent research websites, namely Web of Science, Scopus, and Google Scholar. ECs for other food categories have not been covered here.

## 2. Classification of ECs

### 2.1. Based on Composition

The properties (physical and mechanical) are exclusively dependent on the ingredient of choice. Proteins provide barrier protection that is distinct from that provided by polysaccharides or lipids. Their combination in coatings (composites) may offer a variety of properties. Polysaccharides, proteins, and/or their blends form the base with some included functional ingredients such as antimicrobial agents. In some cases, the coating is made with essential oils (EOs) as the main ingredient and the polysaccharide or (rarely) protein acts as a carrier in the emulsion. The complexity of meat demands a packaging material capable of serving multiple functions at once. Therefore, no single ingredient can satisfy all the purposes and there must be a blend of ingredients to meet the needs of an ideal packaging material. 

#### 2.1.1. Polysaccharide Coatings

Cellulose, native and modified starch, pectins, seaweed extracts (alginates, carrageenan, and agar), gums (acacia, tragacanth, and guar), pullulan, and chitosan are primarily used to make coatings [9]. These compounds impart hardness, crispness, compactness, viscosity, adhesiveness, and gel-forming ability while preventing dehydration, oxidative rancidity, and surface browning. Although some gums have a negative charge, most are neutral [10,11]. Hydrogen bonds play a crucial role in forming films and their final characteristic due to the presence of several hydroxyl and other polar groups in their structures. Negatively charged gums such as alginate, pectin, and carboxymethyl cellulose (CMC) typically have pH-dependent properties [12,13]. Hydrophobicity and poor mechanical strength are the two significant downsides of having a purely polysaccharide-based coating. 

#### 2.1.2. Protein Coatings

Casein, whey protein (concentrate and isolate), collagen, gelatin, egg albumin, corn, soybean, wheat, cottonseed, peanut, and rice are the most popular examples of proteins used for coatings [14,15,16]. Proteins provide good gas barrier properties but poor moisture diffusion resistance. Their ductility is another problem which can be improved by gelatinization of protein. Studies on the effect of denaturation degree on the physical and mechanical properties of the edible film have reported that protein denaturation reduces the vapor and oxygen permeability; however, the protein orientation at the molecular level remains unclear. In contrast, the instability of protein-based films poses risks in package breakage during transportation, handling, and sale, resulting in limited packaging applications [14,17,18]. 

#### 2.1.3. Lipid Coatings 

Lipids for coatings can be divided into vegetable fats and oil, waxes, natural resins, and EOs. However, all of them are not suitable for meat due to the spoilage caused by lipid oxidation. Pure lipids with proteins or polysaccharides as a carrier are a more effective method to obtain gas barrier and moisture barrier properties. This can further improve the color, appearance, texture, aroma, and simultaneously prevent microbial spoilage [19].

### 2.2. Based on Particle Size 

Emulsions can be categorized as macroemulsions (0.1–0.5 μm), nanoemulsions (20–100 nm), and microemulsions (5–50 nm). The particle size of the emulsion droplets influences the properties or behavior of the emulsions. For emulsions to function as a coating, it is crucial to consider the relation of particle size to the release mechanism and the fate of the droplets throughout the storage life. Homogenization devices are best suited to obtain an emulsion of particular particle size. During homogenization, the large droplets undergo size reduction upon applying mechanical sheer force. This yields uniformly distributed droplets in the dispersed medium. Since the emulsion is thermodynamically unstable, surfactants are introduced to reduce the interfacial tension between the dispersed phase and the dispersed medium [20]. This implies that the particle size (influenced by homogenization and the process conditions) impacts the emulsion stability and, thereby, its encapsulation efficiency [21], more of which will be covered in the subsequent sections. An example of this correlation is the study of O/W system with droplet size <200 nm, encapsulating resveratrol. It was found that the emulsion system was stable for 4 weeks while preserving resveratrol from lipid oxidation and its antimicrobial properties [22].

## 3. The Complexity of ECs for Meats: Essential Properties of Consideration

### 3.1. Ingredient Separation

The most common phenomenon disrupting the emulsion is the ingredient separation occurring due to gravitation, coalescence, flocculation, or phase separation. The phases orient themselves according to their concentration and polarity [23,24,25]. Separation may also occur due to environmental changes such as temperature, pressure, and pH. Ingredient separation can be monitored by creaming stability analysis and thermal analysis via DSC; it is also reflected in the microstructure analysis of the coating. At a molecular level, the separation occurs due to the difference in the type of chemical bond that an ingredient can make. Emulsions separate due to the difference in the type of bonds they can make with each other. Water can make strong hydrogen bonds, whereas the oil phase makes only relatively weak van der Waals bonds [26,27]. This creates a thermodynamically unstable environment that can be stabilized by providing free energy to increase the area of interaction between the ingredients. This energy can be estimated by the following equation [28]:

$$\Delta G=\gamma_{i\;}\Delta A$$

where Δ*G* is the free energy required to increase the area of contact between two immiscible liquids by Δ*A* (at constant temperature and pressure) and γ*_i_* is the constant of proportionality called as the interfacial tension.

McClements [23] claims that the magnitude of imbalance in the molecular interaction across the interface determines the interfacial tension; the greater the imbalance, the higher the interfacial tension. In this way, emulsifiers are used to act as a border between the two phases and create uniformity. The most common emulsifiers reportedly used are tween 80 [29,30,31,32] (concentration varying from 0.2–25 wt% of oil/v, tween 20 [30,33,34] (concentration same as tween 80), polyglyceryl-6-dioleate (3–7% *w*/*w*) [35] and lecithin (10% *w*/*v*) [29,36] with tween 80 being the most common one reported. Some researchers have skipped using an emulsifier and instead used a high-velocity homogenizer to obtain a uniform emulsion [37,38]. 

The emulsion’s flow behavior (laminar or turbulent) affects the particle coalescence. The partition and blending of the dispersed phase and dispersed medium to achieve uniform mixing is the first step of homogenization [39,40]. The remaining part of the process involves disruption of larger droplets into smaller ones. Therefore, an understanding of the forces responsible for the droplet disruption during homogenization is important. According to Mc Clements [23] the fate of the droplets formed during homogenization depends on the balance between the interfacial forces holding the droplets together and disruptive forces generated in the homogenizer separating them [41,42].

The literature indicated that emulsions prepared using a homogenizer produced extremely uniform emulsion in the particle size range of 250–2000 nm. Some experiments have also used an ultrasonicator to prepare emulsions; however, the particle sizes were lower than those produced by homogenizers (particle size range: 50–300 nm) [30,43,44,45]. The selection of a homogenizer depends on the desired particle size, the amount of product to be homogenized, the physicochemical properties of the components and the characters required in finished product, and most importantly, the energy costs. Table 2 summarizes the particle size in nanometers (nm) as reported by some of the authors in their studies. Since homogenizers have high energy costs, researchers have tried to achieve similarly stable emulsions using low-energy-cost methods such as spontaneous emulsification, the phase inversion method, the phase inversion temperature method, spontaneous ultrasonication, etc. [46,47]. Despite being low energy methods, they have the major disadvantage of using a high ratio of emulsifier and oil. 

### 3.2. Rheology

The study of an emulsion’s rheological properties is crucial in understanding the protective ability of the coatings for meat or any other food product. The fluidity and spreadability of the emulsions are the most consequential rheological characteristics of consideration. It was surprising for the authors to find that only 13% of research (as per the previously mentioned criteria) conducted rheological studies of the coating emulsions for meat. Since meat has an irregular surface, it becomes highly pertinent to investigate the viscosity and spreadability index of the emulsions. The literature indicated that the coating emulsions manufactured for meat and products exhibited a decrease in viscosity with increasing shear rate and shear thinning properties [44,49,69,70]. Coatings prepared with EOs revealed an increase in apparent viscosity with increasing oil concentrations. It was also observed that the viscosity of the coatings reduced significantly after 30–32 days of storage for the samples prepared with the highest amounts of gelling agent and fat (beeswax and animal fat in a fat blend) [37,70]. 

On a much deeper level, one must understand the rheological behavior of the interfacial layer that surrounds the emulsion droplets [71,72]. According to Murray and Dickenson [73], interfacial rheology is defined as the “study of the mechanical and flow properties of adsorbent layers at fluid interfaces” whereas the stresses responsible for the movement of the interfacial regions (relative to one another) without disruption of overall surface are called interfacial shear deformation. However, they may cause the surface area to expand or contract, which is termed the interfacial dilational deformation [27,74,75]. The interfacial rheology is, consequentially, controlled by the factors (emulsifier concentration, pH, temperature, and ionic strength) that influence the character and strength of the interactions between the molecules absorbed at the interface [76,77]. 

### 3.3. Thermal Stability 

Thermal analysis is not a usual technique chosen by researchers; however, the data give significant results concerning the fate of emulsion droplets and their nature. Emulsion systems are known to be unstable, and therefore they unwind irreversibly into respective bulk phases. However, one can obtain a kinetically stable system that enables analysis via calorimetry, but only if it accompanies the adsorption and release of energy. The primary purpose of thermal analysis is to study the melting and crystallization behavior of emulsion droplets, which is possible by differential scanning calorimetry (DSC) and differential thermal analysis (DTA) [23]. The analysis takes place based on detecting the energy adsorbed (melting) or released (crystallization) by the emulsion systems under the specified temperature conditions. These measurements help monitor the influence of ingredients and experiment conditions on the melting and crystallization behavior of the bulk phases [70,78]. Furthermore, thermal analysis also gives a detailed insight into the polymorphic forms of the triacylglycerols and the glass transition state of the polysaccharides and proteins. It also helps determine the droplets’ stability to coalescence when the bulk phases melt or crystallize; this is because the droplet crystallization temperature is directly proportional to its size owing to the supercooling effects. A droplet’s energy release during crystallization is instantaneous because it occurs far from thermodynamic equilibrium, whereas the energy absorption occurs at the fixed temperature of melting and its kinetics are determined by interactions with the medium surrounding it [79]. 

### 3.4. Antioxidant and Antimicrobial Properties

Essential requirements for ECs in enhancing the shelf life of meat and products are antimicrobial and antioxidant properties. The importance of discussing the parameters of the aforementioned properties lies in the fact that they influence the efficacy of ECs for meat considerably and ECs without them are rendered less effective. As previously established, the high susceptibility of meat to spoilage demands that its shelf life be considered the highest priority.

Since fresh meat is highly susceptible to oxidation (lipid and protein), it is one of the major parameters of concern. Hydroperoxides are produced through lipid peroxidation, and when these compounds are broken down, secondary oxidative products are produced, resulting in unpleasant odors and flavors in meat [32,80,81]. Oxidative damages due to uncontrolled formation of free radicals result in quality decay, loss of flavor, texture, color, and nutritive values of the meat due to PUFA (polyunsaturated fatty acids) degrading [1,82,83,84]. Natural antioxidants from plants and extracts thereof have been of interest to researchers; however, ECs’ effectiveness against lipid and protein oxidation has not yet been studied extensively. Natural antioxidants prevent the formation and spread of reactive species and free radicals by acting as hydrogen donors and scavengers of free radicals [85]. 

**Step 1 Initiation:** Heat, metal ions, and irradiation act as catalysts and form lipid free radicals that react with oxygen to produce peroxy radicals.

(1) $$R+O_{2}\to \;R•+•\mathrm{OOH}$$

**Step 2 Propagation:** Peroxide radicals react with other lipid molecules to produce hydroperoxides and more free radicals as follows:

(2) $$R•+O_{2}\to \;\mathrm{ROO}•$$

(3) RH+ROO•→ ROOH+R•

(4) ROOH→ RO•+•OH

**Step 3 Termination:** Reaction between two free radicals results in the termination of the reaction.

(5) R•+R•→  R–R

(6) R+ROO•→ ROOR

(7) $$\mathrm{ROO}•+\mathrm{ROO}•\to \;\mathrm{ROOR}+O_{2}$$

Lobo et al. [84] explained the three distinct methods of oxidative protein modification: through a specific amino acid’s oxidative modification; by breaking the peptide caused by free radicals; and by the reaction with the products of lipid peroxidation, which results in the formation of the transverse binding protein. The protein’s susceptibility to oxidation and enzymatic proteolysis increases with the presence of amino acids such as cysteine, histidine, methionine, and arginine, which are modified by free radicals [86,87,88,89]. Carbonyl formation is a common reaction pathway in the oxidation process, and the same oxidants that start lipid oxidation also cause and propagate protein oxidation. Carbonyl derivatives and protein–lipid and protein–protein complexes are also produced when proteins react with secondary lipid peroxidation products such as ketones and aldehydes [90,91,92,93]. •OH is readily produced in meats when hydrogen peroxide or lipid peroxide reacts with copper or iron to modify amino acids such as lysine and methionine in specific locations.

H_2_O_2_ + Fe(II)/Cu(I) → •OH + OH^−^ + Fe(III)/Cu(II) (8)

•OH + Protein (lysine)-NH_2_
→ Protein–COH (carbonyl) (9)

Currently the EOs from plants, their extracts, and by-products are gaining much attention. Typically, EOs are a source of phenolic and polyphenolic substances with potent antioxidant properties [92]. The functional properties of emulsions can be improved by the synergistic effects of the EOs and their constituents, which in turn can successfully extend the shelf life of meat, particularly that high in fat such as pork. Due to the strong radical scavenging properties of phenolic compounds, the antioxidant activity of EOs is associated with mechanisms such as interaction with free radicals, hydroperoxide decomposition, inhibition of chain reactions, and transitional binding of metals [93,94]. The thermodynamic stability of phenolic radicals can be attributed to their resonant structures, according to Majdinasab [32]. This implies that the CHO fraction of EOs is a more effective antioxidant than the phenolic content [84]. 

The oxidation extent can be evaluated by peroxide value (PV) determination or by the thiobarbituric acid reactive substances (TBARS) method. In a study, shrimp samples treated with 3% and 5% saffron EO emulsions were found to have significantly low PV in comparison to the untreated samples, especially at the end of the 14th day of the study. A TBARS assay also revealed a huge difference in the TBA value of the emulsion coated samples and the uncoated ones [34]. Similar results were discovered in a study on nanoemulsion prepared from oregano EO and resveratrol in pectin EC. It was found that the coated pork loin samples reached close to the threshold spoilage value (4.20 mg MDA (malonaldehyde)/kg) on day 20 of the study, whereas the uncoated samples crossed the threshold value on the 5th day of study [31]. Emulsions prepared from lipopeptides and BHA in sunflower coating for raw beef patties revealed that the TBARS value of the uncoated samples increased rapidly on storage and reached a value of 2.8 mg MDA/kg on the 12th day of storage. The value was much lower for the emulsion-coated samples (0.35 and 0.25 for lipopeptides and BHA coating, respectively) [33]. The TBARS value of chicken meat coated with rosemary extract and ε-poly-L-lysine was found to be significantly lower (1.523–1.97 mg MDA/kg) than that of the uncoated samples (2.27 mg MDA/kg) [48]. Chicken fillets coated with BSG-based coatings had a peroxide value of 5 meq active O_2_/kg lipid after 12 days whereas the uncoated samples had a value of 8.07 meq active O_2_/kg lipid. The authors reported that the formation of hydroperoxides was low due to the antioxidant properties of thyme EO and summer savory EO. They attributed this to thyme’s higher amounts of phenolic compounds, such as carvacrol and thymol, and their synergistic effect with other trace components in thyme EO [32]. Chicken meat coated with camellia oil-loaded EC was found to have a TBARS value of 0.695 mg MDA/kg on the 10th day whereas the uncoated sample had a value of 1.824 mg MDA/kg. The authors defended the results by stating that the active compound in the camellia oil was most likely to be responsible for scavenging the free radicals, chelation of metal ions, inhibition of lipid peroxidation, and regulation of the antioxidant enzymes’ levels [50]. 

Winther et al. [95] determined the thiol groups in pork samples to check for protein oxidation. Cystein and other amino acids’ thiol group can easily oxidize to form disulfide bonds, reducing the thiol. A peroxide oxidation study conducted by Xiong [31] on pork loin coated in oregano EO and resveratrol emulsion in pectin revealed that the thiol group value on day 0 was 57.87 nmol thiol/mg. These values reduced significantly for the uncoated samples, indicating high oxidation, whereas the value was significantly higher for the oregano EO and resveratrol-coated samples. In another study, the content of TVN (total volatile nitrogen) determined by the Kjeldahl method was used to track the protein peroxidation. In the control group, the TVN of the meat showed a trend of rapid increase, reaching 44.516 mg/100 g on the tenth day. This was the maximum level that was considered passable in rainbow trout fillets. However, on day 10, the treated samples increased to 21.549 mg/100 g. TVN was mostly produced when protein and other non-protein nitrogenous compounds were broken down by the bacteria [96]. Coating’s microbial inhibitory effects and the coating’s function as a physical barrier to keep oxygen out of meat samples were the main reason for low TVN formation in coated samples. According to Majdinasab [32], compared to the control samples, the EC made from basil-seed gum containing summer savory EO oil and shirazi thyme EO significantly reduced TVN values. Ojagh et al. [96] discovered that the lower TVN values of samples coated with polyphenol-rich EOs were due to the reduced bacterial count or their incapacity for protein decomposition [50]. 

ECs developed with a variety of EOs and their blends are also highly efficient as antimicrobial agent (due to compounds such as terpenoids, terpenes, and aliphatic chemicals) against a variety of microbial species. For meat, the important microorganisms of concern are *Pseudomonas* spp., *Enterobacteriaceace*, *Staphylococcus* spp., lactic acid bacteria (LAB), yeasts, and molds. Table 2 summarizes the emulsion compositions, antimicrobial/antioxidant agents, and target bacterial strains tested. 

Majority of the literature indicated the preference for EOs as a suitable antimicrobial agent. Only a few studies used ingredients such as vitamin C, tea polyphenols, duck fat, daphnetin, and some plant extracts. ECs developed with EOs indicated an enhanced shelf life for up to 15 days and in some cases as much as 126 days. An exception that was included in the review was an EC developed for eggs using beeswax and basil EO. The shelf life of eggs at room temperature was studied against *E-coli* and *S. aureus* at room temperature for 32 days. This can be attributed to the high variety of polyphenols present in the oils. According to Serra et al. and Puuppnen [97,98], mixtures of polyphenols from plant extracts have higher influence on the microbial activity than the individual compounds. This is supported by the fact that there is a positive synergistic effect between the polyphenols against the microbial activity [99].

In addition to this, it is worth mentioning that the efficiency of antimicrobial agents in increasing shelf life highly depend on the particle size of the emulsion, type of material used for encapsulation, and the mechanism of sustained release, the latter being topics not thoroughly researched for ECs. 

## 4. Effects of EC on Meat Quality

Adequate literature exists proving the advantages of ECs for meat and related products. The major highlight is the ability of ECs to extend the shelf life of the meat to 12–126 days. As previously established, the rapid microbial spoilage of meat, especially by *Listeria monocytogenes*, is a grave concern to the meat industry owing to bacteria’s ability to form a mucilaginous coating. The incorporation of EOs is highly beneficial to combat this situation. It was successfully demonstrated by Catarino et al. [16] in their study on oregano-loaded EC in Portuguese sausage. The shelf life of the sausages extended to 106–126 days under refrigeration. Similar results were reported by Kazemeini et al. [52] in their study of EC loaded with Trachyspermum ammi EO on turkey fillets. 

The appearance of meat is the most important parameter for consumers’ assessment of freshness and quality. The color, texture, and moisture content are highly regarded in validating the freshness. The gas barrier and lipid and protein oxidation-prevention properties of ECs are mainly responsible for the color preservation of meat. ECs-coated meat has been reported to retain an appealing color on day 8 of study: Noori et al. [30] reported excellent results in their research on nanoemulsions loaded with ginger EO. They found that the color change was directly proportional to the concentration of EO in their emulsions. Furthermore, the nanoemulsions also reduced the rate of odor degradation. 

The hardness and tenderness of meat depend on the moisture content of the meat. It also varies to a considerable extent depending on protein degradation. Pork loin samples as analyzed by Xiong et al. [31] revealed that the EC fabricated with oregano EO and resveratrol had a considerably better texture than the control samples. This was because of the capacity of the film to prevent protein degradation. Additionally, the nanoemulsions had even better performance than the conventional emulsions.

## 5. Limitations of the Work

Although the number of studies we examined was sufficient, there was a lack of information regarding the fate of the coatings during and after cooking. A comparative study on the physical properties of the emulsions prepared using different homogenizers would be an attractive research aspect. The method of emulsion preparation, the study of physical properties, and their correlation with its performance would also be interesting topics to be explored. It was also observed that none of the research, as per our knowledge, covered the antioxidant release mechanism of ECs. Since the efficacy of ECs as a suitable antioxidant/antimicrobial agent depends heavily on their release rate, it is vital to study and understand the mechanism. Furthermore, there needs to be sufficient data on the types of emulsions, such as nanoemulsions, nanoencapsulation, multilayer emulsions, and others that are more stable and suitable than the conventional O/W or W/O types. Additionally, to assess the efficacy of the coatings for meat, it is fundamental to study lipid and protein oxidation and the release of antimicrobials/antioxidants during storage. In this sense, multilayer or nanoemulsions might prove more efficient as the new generation of active packaging for meat and related products. However, while some papers state the superiority of nanoemulsions over conventional emulsions, the data are still insufficient and must be explored further.

## 6. Conclusions

ECs have immense potential and have gained much attention in the past decade, especially for meat. Their ease of application is highly suitable for irregularly shaped products such as poultry breast pieces and pork loin. However, their construction comes with a challenge, and combining multiple properties in one coating is required to provide holistic protection. While ECs featuring physical stability and antioxidant/antimicrobial agents have been studied thoroughly, numerous research gaps need to be explored and evaluated to improve the application of the coatings. Novel methodologies focusing on physical attributes such as rheology, thermal stability, encapsulation, sensory attributes, periodic/gradual release of antimicrobials agent, and cooking stability are required to develop a new line of edible packaging suitable for consumers. 

## Figures and Tables

**Table 1:** Composition of meat from different animals.

Meat	Nutritional Composition (per 100 g)				Energy (kJ/100 g)	References
	Water	Protein	Fat	Ash		
Beef (lean)	75.0	22.3	1.8	1.2	485	[2]
Beef carcass	54.7	16.5	28.0	0.8	1351	
Pork (lean)	75.1	22.8	1.2	1.0	469	
Pork carcass	41.1	11.2	47.0	0.6	1975	
Veal (lean)	76.4	21.3	0.8	1.2	410	
Chicken	75.0	22.8	0.9	1.2	439	
Mutton carcass	73.9	20.2	4.86	1.18	524	[3]
Chevon carcass	75.6	20.3	3.68	4.09	-	[4]
Buffalo carcass	76.3	20.4	1.37	0.98	724	[5]

**Table 2:** Summary of the composition, particle size, antimicrobial/antioxidant compound, target microorganisms, production, and shelf life conditions studied in the literature.

Product	Coating Material	Particle Size (nm)	Antimicrobial/Antioxiadnt Compound	Target Microorganisms	Conditions	Reference
Chicken	Gelatin and chitosan nanoemulsion coating	1122.4	Rosemary extract in corn germ oil and ε-poly-_L_-lysine	Coliforms, *E-coli*, molds, yeast	4 °C, 16 days (d) Coated by soaking, covered with cling film and stored	[43]
Eggs	Chitosan	1483–983	Beeswax-basil EO	*E-coli, S. aureus*	Room temperature, 35 d 2 mL coating sprayed and dried.	[44]
Turkey breast fillets	Chitosan	342–5149	*Zataria Multiflora Boiss* EO and *Bunium persicum Boiss* EO	*Salmonella enteritidis*, *Listeria monocytogenes*, TVC (total viable count), total *Pseudomonas* spp., *Enterobacteriaceae*, LAB (lactic acid bacteria), and yeast and mold count	4 °C, 18 d Coated with nano-emulsion for 2 min, drained for 1 h, and packed in zip lock bags.	[29]
Chicken breast fillet	Sodium caseinate	57.4	Ginger EO	*Listeria monocytogenes* and *Salmonella typhimurium*	4 °C, 12 d Coating by direct immersion and packed in LDPE	[30]
Raw goat meat	Gum arabic	220–260	geraniol and carvacrol	*Bacillus cereus* and *E-coli*	4 °C, 9 d Coating by direct dipping.	[45]
Pork loin	Pectin	48.5–335.9	Oregano EO and resveratrol	TVC	4 °C, 20 d Coating by direct immersion for 30 s, air drying, and sealed hermetically in plastic trays with 20% CO_2_ and 80% O_2._	[31]
Shrimps	Oil-in-water nanoemulsion	10.2–11	Saffron EO	*E-coli* and *S aureus*	4 °C and 8 °C 14 d Coating by direct immersion, draining, and sealing in PE (polyethylene) bags.	[34]
Red sea bream	Oil-in-water nanoemulsion	799.5–114.7	Ginger EO	*E-coli* and *S aureus*	4 °C, 10 d Coating by direct dipping.	[48]
Fresh pork tenderloin	Gelatin	4000–6000	Eugenol EO	TVC, *Enterobacteriaceae* lactic acid bacteria, *Pseudomonas* spp.	4 °C, 15 d Coating by soaking for 30 s and covered with cling films.	[49]
Chicken fillets	Basil seed gum	-	Shirazi thyme EO and summery savory EO	Mesophilic, psychrotrophic and LAB	4 °C, 12 d Coating by soaking for 120 s, drain for 2 min ×2.	[32]
*Scopthalmus Maximus*	Locust bean gum and sodium alginate	-	Daphnetin	TVC, psychrophiles, and *Pseudomonas* spp.	4 °C, 18 d Coating by direct dipping for 20 min at 4 °C, drying for 60 min in air flow at 4 °C and then individually packed in PE.	[36]
Chicken fillet	Konjac glucomannan/carrageenan	-	Camellia EO	TVC, psychrophiles, and LAB	4 °C, 10 d Coating by direct immersion for 10 s, drying at ambient temp. for 10 min, and covering with plastic wrap.	[50]
Turkey fillets	Alginate	156.2	*Trachyspermum ammi* EO	*Listeria monocytogenes*	4 °C, 12 d Coating by direct dipping, dipping in 2% CaCl_2_ solution for 30 s and, packed in sterile zipper packs.	[51]
Sliced bolognas	Pectin	-	*Thymus vulgaris* and *Thymbra spicata* EO	Mesophilic and LAB	4 °C, 21 d Coating by dipping for 2 min and draining for 3 min before storage.	[52]
Pork meat	Chitosan	-	Thyme EO	*Pseudomonas*, *Lactococcus,* and *Acinetobacter*	4 °C, 12 d Coating by alcohol spraying, air drying for 5 min, and packed in plastic bags.	[53]
Ready to eat chicken patties	Chicken bone gelatin–chitosan	1370.8–183.6	Cinnamon EO and rosemary extract	*E. coli*, *Bacillus subtilis,* and *S aureus*	4 °C, 16 d Coating by direct dipping for 3 min, drained for 10 min and sealed in PE bags.	[38]
Crayfish meat	Chitosan	-	Propolis extract	Total aerobic mesophilic, psychrotrophic and H_2_S-producing bacteria, yeasts- molds	4 °C, 16 d Coating by direct dipping for 2 min, drying for 60 min in air flow at 10 °C and then individually packed in sterile PE.	[54]
Chicken meat	Chitosan	-	Duck fat	TVC and *Listeria* spp.	4 °C, 15 d Coating by direct dipping for 2 min under magnetic stirring at 800 rpm, dried in laminar hood at 25 °C for 2 h and packed in PE bags.	[55]
Pork	Chitosan	389.7–45.3	*Schizonepeta tenuifolia* EO	TVC, *Pseudomonas* spp., LAB, and *Enterobacteriaceae*	4 °C, 16 d Coating by direct dipping for 30 s) ×2 with 2 min break, draining for 10 min, and packed in oxygen-permeable PE film.	[56]
Fresh meat	Chitosan	>1000	Eugenol EO	*E. coli* and *S. aureus*	4 °C, 14 d Coating by direct dipping for 1 min and draining.	[57]
Chicken breast	Calcium alginate	-	*Artemisia fragrance* EO	TVC, coliforms, molds and yeast	4 °C, 12 d Coating by direct dipping for 60 min at 4 °C, draining and packing in PE bags.	[58]
Silver carp fillet	Sodiumalginate–carboxymethyl cellulose	-	*Ziziphora clinopodioides* EO, apple peel extract, and zinc oxide nanoparticles (alone and in combination)	*Listeria monocytogenes*	4 °C, 14 d Coating by direct immersion at room temperature for 30 s, drained for 15 min, and dried under refrigeration for 2 h.	[59]
Chicken breast	Whey protein isolate	-	Oregano and clove EO	Total aerobic mesophilic bacteria, *Enterobacteriaceae*, total aerobic psychrotrophic bacteria, LAB, and *Pseudomonas* spp.	4 °C, 13 d Coating by immersion, draining, and drying under sterilized conditions.	[15]
Paínho and alheira Portuguese sausage	Whey protein	-	*Origanum virens* EO	*Salmonella* spp. And *L. monocytogenes*	4 °C, 106–126 d Coating by applying 1 mL of emulsion by silicone brush and packing in LDPE films by thermal vacuum sealing (30 s at 120 °C)	[16]
Chicken breast	Pomegranate juice–chitosan	-	*Zataria multiflora EO*	TVC, *Pseudomonas* spp., lactic acid bacteria, *Enterobacteriaceae*, Psychrotrophic bacteria and yeasts– molds	4 °C, 20 d Coating by direct immersion for 2 min twice with a short interval, drained for 5 h at 10 °C, and packing in sterilized LDPE packages.	[60]
Rainbow trout fillet	Chitosan	-	*Mentha spicata* EO	TVC, psychrotrophic bacteria, *Pseudomonas* spp. and *Enterobacteriaceae*	4 °C, 14 d Coating by direct immersion for 1 min, draining for 5 min, and packing in sterile stomacher bags.	[61]
Silver carp fillet	Methylcellulose	-	*Pimpinella affinis* EO	TVC and psychrotrophic bacteria	4 °C, 20 d Coating by direct immersion for 30 s ×2, and drained for 5 h at 10 °C.	[62]
Bighead carp fillet	Sodium Alginate	-	Horsemint (*Mentha longifolia)* EO	TVC and psychrotrophic bacteria	4 °C, 16 d Coating by direct immersion for 30 s and drained for 30 min at ambient conditions,	[63]
Rainbow trout fillet	Fish gelatin	-	Oregano EO	TVC	4 °C, 16 d Coating by direct immersion for 2 min ×2 with 1 min draining interval and drying for 1 h under sterile laminar hood.	[64]
Shrimp	Chitosan	-	Garlic EO	Aerobic plate count	4 °C, 11 d Coating by direct immersion for 5 min, drained and dried for 4 h at 4 °C, and packed in plastic wrap.	[65]
Lamb meat	Chitosan	96–93	*Satureja* plant EO	TVC, *Pseudomonas* spp. and LAB	4 °C, 20 d Coating by direct immersion for one min) ×2, drained and dried for 15 min at 25 °C	[66]
Rainbow trout fillet	Carboxymethyl cellulose	-	*Zataria multiflora Boiss* EO and grapeseed extract	TVC, *Pseudomonas* spp. and LAB	4 °C, 20 d Coating by direct immersion and drained.	[67]
Refrigerated bream (*Megalobrama amblycephala)*	Sodium alginate	-	Vitamin C and tea polyphenols	TVC	4 °C, 20 d Coating by direct immersion for 1 min, air dried for 1 min, and immersed in CaCl_2_ and packed in PE bags.	[68]
Trout (Oncorhync-husmykiss) fillets	Carrageenan	-	Lemon EO	TVC, *Pseudomonas* spp. and *Enterobacteriaceae*	4 °C, 15 d Coating by direct immersion.	[69]

## Data Availability

The data presented in this study are available on request from the corresponding author.

## References

[1] Zhou G.H., Xu X.L., Liu Y. (2010). Preservation technologies for fresh meat—A review. Meat Sci..

[2] Heinz G., Hautzinger P. (2007). Meat Processing Technology for Small to Medium Scale Producers.

[3] Sainsbury J., Schönfeldt H.C., Van Heerden S.M. (2011). The nutrient composition of South African mutton. J. Food Compos. Anal..

[4] Soren N.M., Biswas A.K. (2020). Methods for Nutritional Quality Analysis of Meat.

[5] Naveena B.M., Kiran M., Reddy K.S., Ramakrishna C., Vaithiyanathan S., Devatkal S.K. (2011). Effect of ammonium hydroxide on ultrastructure and tenderness of buffalo meat. Meat Sci..

[6] Tauro P., Kapoor K.K., Yadav K.S. (1986). An Introduction to Microbiology.

[7] Cheng L.N., Sun D.W., Zhu Z.W., Zhang Z. (2017). Emerging techniques for assisting and accelerating food freezing processes: A review of recent research progresses. Crit. Rev. Food Sci. Nutr..

[8] Chivandi E., Dangarembizi R., Nyakudya T.T., Erlwanger K.H. (2016). Chapter 8—Use of Essential Oils as a Preservative of Meat.

[9] Murtaja Y., Lapčík L., Lapčíková B., Gautam S., Vašina M., Spanhel L., Vlček J. (2022). Intelligent high-tech coating of natural biopolymer layers. Adv.Colloid Interface Sci..

[10] Eroglu E., Torun M., Dincer C., Topuz A. (2014). Influence of Pullulan-Based Edible Coating on Some Quality Properties of Strawberry During Cold Storage. Packag. Technol.Sci..

[11] Gennadios A., Hanna M.A., Kurth L.B. (1997). Application of edible coatings on meats, poultry and seafoods: A review. Food Sci. Technol..

[12] Dehghani S., Hosseini S.V., Regenstein J.M. (2018). Edible films and coatings in seafood preservation: A review. Food Chem..

[13] Dong J., Kou X., Liu L., Hou L., Li R., Wang S. (2021). Effect of water, fat, and salt contents on heating uniformity and color of ground beef subjected to radio frequency thawing process. Innov. Food Sci. Emerg. Technol..

[14] Sanchez-Ortega I., Garica-Almendarez B.E., Santos-Lopez E.M., Amaro-Reyes A., Barboza-Corona J.E., Regalado C. (2014). Antimicrobial Edible Films and Coatings for Meat and Meat Products Preservation. Sci. World J..

[15] Fernández-Pan I., Carrión-Granda X., Maté J.I. (2014). Antimicrobial efficiency of edible coatings on the preservation of chicken breast fillets. Food Control.

[16] Catarino M.D., Alves-Silva J.M., Fernandes R.P., Gonçalves M.J., Salgueiro L.R., Henriques M.F., Cardoso S.M. (2017). Development and performance of whey protein active coatings with Origanum virens essential oils in the quality and shelf life improvement of processed meat products. Food Control.

[17] Schmid M., Krimmel B., Grupa U., Noller K. (2014). Effects of thermally induced denaturation on technological-functional properties of whey protein isolate-based films. J. Dairy Sci..

[18] Guckian S., Dwyer C., O’Sullivan M., O’Riordan E.D., Monahan F.J. (2006). Properties of and mechanisms of protein interactions in films formed from different proportions of heated and unheated whey protein solutions. Eur. Food Res. Technol..

[19] Cutter C.N. (2006). Opportunities for bio-based packaging technologies to improve the quality and safety of fresh and further processed muscle foods. Meat Sci..

[20] Liu B., Hu X. (2020). Hollow Micro-and Nanomaterials: Synthesis and Applications.

[21] Lu W., Kelly A.L., Miao S. (2016). Emulsion-based encapsulation and delivery systems for polyphenols. Trends Food Sci.Technol..

[22] Donsì F., Sessa M., Mediouni H., Mgaidi A., Ferrari G. (2011). Encapsulation of bioactive compounds in nanoemulsion-based delivery systems. Procedia Food Sci..

[23] McClements D.J. (2004). Food Emulsions: Principles, Practices, and Techniques.

[24] Chanamai R., Horn G., McClements D.J. (2002). Influence of oil polarity on droplet growth in oil-in-water emulsions stabilized by a weakly adsorbing biopolymer or a nonionic surfactant. J. Colloid Interface Sci..

[25] Christenson H.K., Per M. (2001). Claesson. Direct measurements of the force between hydrophobic surfaces in water. Adv. Colloid Interface Sci..

[26] Norde W. (2011). Colloids and Interfaces in Life Sciences and Bionanotechnology.

[27] Dickinson E. (2003). Hydrocolloids at interfaces and the influence on the properties of dispersed systems. Food Hydrocoll..

[28] Hiemenz P.C., Rajagopalan R. (2016). Principles of Colloid and Surface Chemistry, Revised and Expanded.

[29] Keykhosravy K., Khanzadi S., Hashemi M., Azizzadeh M. (2020). Chitosan-loaded nanoemulsion containing Zataria Multiflora Boiss and Bunium persicum Boiss essential oils as edible coatings: Its impact on microbial quality of turkey meat and fate of inoculated pathogens. Int. J. Biol. Macromol..

[30] Noori S., Zeynali F., Almasi H. (2018). Antimicrobial and antioxidant efficiency of nanoemulsion-based edible coating containing ginger (Zingiber officinale) essential oil and its effect on safety and quality attributes of chicken breast fillets. Food Control.

[31] Xiong Y., Li S., Warner R.D., Fang Z. (2020). Effect of oregano essential oil and resveratrol nanoemulsion loaded pectin edible coating on the preservation of pork loin in modified atmosphere packaging. Food Control.

[32] Majdinasab M., Niakousari M., Shaghaghian S., Dehghani H. (2020). Antimicrobial and antioxidant coating based on basil seed gum incorporated with Shirazi thyme and summer savory essential oils emulsions for shelf-life extension of refrigerated chicken fillets. Food Hydrocoll..

[33] Jemil N., Ouerfelli M., Almajano M.P., Elloumi-Mseddi J., Nasri M., Hmidet N. (2020). The conservative effects of lipopeptides from Bacillus methylotrophicus DCS1 on sunflower oil-in-water emulsion and raw beef patties quality. Food Chem..

[34] Aboutorab M., Ahari H., Allahyaribeik S., Yousefi S., Motalebi A. (2021). Nano-emulsion of saffron essential oil by spontaneous emulsification and ultrasonic homogenization extend the shelf life of shrimp (*Crocus sativus* L.). J. Food Process. Preserv..

[35] Yuan D., Hao X., Liu G., Yue Y., Duan J. (2022). A novel composite edible film fabricated by incorporating W/O/W emulsion into a chitosan film to improve the protection of fresh fish meat. Food Chem..

[36] Liu W., Mei J., Xie J. (2021). Effect of locust bean gum-sodium alginate coatings incorporated with daphnetin emulsions on the quality of Scophthalmus maximus at refrigerated condition. Int. J. Biol. Macromol..

[37] Kowalska M., Babut M., Woźniak M., Żbikowska A. (2019). Formulation of oil-in-water emulsions containing enzymatically modified rabbit fat with pumpkin seed oil. J. Food Process. Preserv..

[38] Qiu L., Zhang M., Chitrakar B., Adhikari B., Yang C. (2022). Effects of nanoemulsion-based chicken bone gelatin-chitosan coatings with cinnamon essential oil and rosemary extract on the storage quality of ready-to-eat chicken patties. Food Packag. Shelf Life.

[39] Seekkuarachchi I.N., Tanaka K., Kumazawa H. (2006). Formation and charaterization of submicrometer oil-in-water (O/W) emulsions, using high-energy emulsification. Ind. Eng. Chem. Res..

[40] Walstra P. (1993). Principles of emulsion formation. Chem. Eng. Sci..

[41] Fischer P., Erni P. (2007). Emulsion drops in external flow fields—The role of liquid interfaces. Current Opin. Colloid Interface Sci..

[42] Williams A., Janssen J., Prins A. (1997). Behaviour of droplets in simple shear flow in the presence of a protein emulsifier. Colloids Surf. Physicochem. Eng. Aspects.

[43] Huang M., Wang H., Xu X., Lu X., Song X., Zhou G. (2020). Effects of nanoemulsion-based edible coatings with composite mixture of rosemary extract and ε-poly-L-lysine on the shelf life of ready-to-eat carbonado chicken. Food Hydrocoll..

[44] Sun R., Song G., Zhang H., Zhang H., Chi Y., Ma Y., Li H., Bai S., Zhang X. (2021). Effect of basil essential oil and beeswax incorporation on the physical, structural, and antibacterial properties of chitosan emulsion based coating for eggs preservation. LWT.

[45] Syed I., Banerjee P., Sarkar P. (2020). Oil-in-water emulsions of geraniol and carvacrol improve the antibacterial activity of these compounds on raw goat meat surface during extended storage at 4 C. Food Control.

[46] Santana R.C., Perrechil F.A., Cunha R.L. (2013). High-and low-energy emulsifications for food applications: A focus on process parameters. Food Eng. Rev..

[47] Saberi A.H., Fang Y., McClements D.J. (2013). Effect of glycerol on formation, stability, and properties of vitamin-E enriched nanoemulsions produced using spontaneous emulsification. J.Colloid Interface Sci..

[48] Cai L., Wang Y., Cao A. (2020). The physiochemical and preservation properties of fish sarcoplasmic protein/chitosan composite films containing ginger essential oil emulsions. J. Food Process. Eng..

[49] Wan J., Pei Y., Hu Y., Ai T., Sheng F., Li J., Li B. (2020). Microencapsulation of eugenol through gelatin-based emulgel for preservation of refrigerated meat. Food Bioprocess Technol..

[50] Zhou X., Zong X., Zhang M., Ge Q., Qi J., Liang J., Xu X., Xiong G. (2021). Effect of konjac glucomannan/carrageenan-based edible emulsion coatings with camellia oil on quality and shelf-life of chicken meat. Int. J. Biol. Macromol..

[51] Kazemeini H., Azizian A., Adib H. (2021). Inhibition of Listeria monocytogenes growth in turkey fillets by alginate edible coating with Trachyspermum ammi essential oil nano-emulsion. Int. J. Food Microbiol..

[52] Gedikoğlu A. (2022). The effect of Thymus vulgaris and Thymbra spicata essential oils and/or extracts in pectin edible coating on the preservation of sliced bolognas. Meat Sci..

[53] Wang L., Liu T., Liu L., Liu Y., Wu X. (2022). Impacts of chitosan nanoemulsions with thymol or thyme essential oil on volatile compounds and microbial diversity of refrigerated pork meat. Meat Sci..

[54] Çoban M.Z. (2021). Effectiveness of chitosan/propolis extract emulsion coating on refrigerated storage quality of crayfish meat (Astacus leptodactylus). CyTA-J. Food.

[55] Shin D., Kim Y.-J., Yune J.-H., Kim D.H., Kwon H.C., Sohn H., Han S.G., Han J.H., Lim S.J., Han S.G. (2022). Effects of Chitosan and Duck Fat-Based Emulsion Coatings on the Quality Characteristics of Chicken Meat during Storage. Foods.

[56] Zhang H., Li X., Kang H., Peng X. (2021). Antimicrobial and antioxidant effects of edible nanoemulsion coating based on chitosan and Schizonepeta tenuifolia essential oil in fresh pork. J. Food Process. Preserv..

[57] Zhao R., Zhang Y., Chen H., Song R., Li Y. (2022). Performance of eugenol emulsion/chitosan edible coating and application in fresh meat preservation. J. Food Process. Preserv..

[58] Alirezalu K., Moazami-Goodarzi A.H., Roufegarinejad L., Yaghoubi M., Lorenzo J.M. (2022). Combined effects of calcium-alginate coating and Artemisia fragrance essential oil on chicken breast meat quality. Food Sci. Nutr..

[59] Rezaei F., Shahbazi Y. (2018). Shelf-life extension and quality attributes of sauced silver carp fillet: A comparison among direct addition, edible coating and biodegradable film. LWT.

[60] Bazargani-Gilani B., Aliakbarlu J., Tajik H. (2015). Effect of pomegranate juice dipping and chitosan coating enriched with Zataria multiflora Boiss essential oil on the shelf-life of chicken meat during refrigerated storage. Innov. Food Sci. Emerg. Technol..

[61] Shahbazi Y., Shavisi N. (2018). Chitosan coatings containing *Mentha spicata* essential oil and zinc oxide nanoparticle for shelf life extension of rainbow trout fillets. J. Aquat. Food Prod. Technol..

[62] Ariaii P., Tavakolipour H., Rezaei M., Rad A.H.E., Bahram S. (2015). Effect of methylcellulose coating enriched with Pimpinella affinis oil on the quality of silver carp fillet during refrigerator storage condition. J. Food Process. Preserv..

[63] Heydari R., Bavandi S., Javadian S.R. (2015). Effect of sodium alginate coating enriched with horsemint (*Mentha longifolia*) essential oil on the quality of bighead carp fillets during storage at 4 °C. Food Sci. Nutr..

[64] Hosseini S.F., Rezaei M., Zandi M., Ghavi F.F. (2016). Effect of fish gelatin coating enriched with oregano essential oil on the quality of refrigerated rainbow trout fillet. J. Aquat. Food Prod. Technol..

[65] Aşik E., Candoğan K. (2014). Effects of chitosan coatings incorporated with garlic oil on quality characteristics of shrimp. J. Food Qual..

[66] Pabast M., Shariatifar N., Beikzadeh S., Jahed G. (2018). Effects of chitosan coatings incorporating with free or nano-encapsulated Satureja plant essential oil on quality characteristics of lamb meat. Food Control.

[67] Raeisi M., Tajik H., Aliakbarlu J., Mirhosseini S.H., Hosseini S.M.H. (2015). Effect of carboxymethyl cellulose-based coatings incorporated with Zataria multiflora Boiss. essential oil and grape seed extract on the shelf life of rainbow trout fillets. LWT-Food Sci. Technol..

[68] Song Y., Liu L., Shen H., You J., Luo Y. (2011). Effect of sodium alginate-based edible coating containing different anti-oxidants on quality and shelf life of refrigerated bream (*Megalobrama amblycephala*). Food Control.

[69] Volpe M.G., Siano F., Paolucci M., Sacco A., Sorrentino A., Malinconico M., Varricchio E. (2015). Active edible coating effectiveness in shelf-life enhancement of trout (Oncorhynchusmykiss) fillets. LWT-Food Sci. Technol..

[70] Clausse D. (2010). Differential thermal analysis, differential scanning calorimetry, and emulsions. J. Therm. Anal. Calorim..

[71] Derkach S.R. (2009). Rheology of emulsions. Adv.Colloid Interface Sci..

[72] Karbaschi M., Lotfi M., Krägel J., Javadi A., Bastani D., Miller R. (2014). Rheology of interfacial layers. Current Opin. Colloid Interface Sci..

[73] Murray B.S., Dickinson E. (1996). Interfacial rheology and the dynamic properties of adsorbed films of food proteins and surfactants. Food Sci. Technol. Int. Tokyo.

[74] Bos M.A., Van Vliet T. (2001). Interfacial rheological properties of adsorbed protein layers and surfactants: A review. Adv.Colloid Interface Sci..

[75] Benjamins J., Lucassen-Reynders E.H., Miller R., Liggieri L. (2009). Interfacial Rheology of Adsorbed Protein Layers.

[76] Javadi A., Mucic N., Karbaschi M., Won J.Y., Lotfi M., Dan A., Ulaganathan V., Gochev G., Makievski A.V., Kovalchuk V.I. (2013). Characterization methods for liquid interfacial layers. Eur. Phys. J. Special Topics.

[77] Sagis L.M., Scholten E. (2014). Complex interfaces in food: Structure and mechanical properties. Trends Food Sci. Technol..

[78] Atkins P., Atkins P.W., de Paula J. (2014). Atkins’ Physical Chemistry.

[79] Kousksou T., Jamil A., Gibout S., Zeraouli Y. (2009). Thermal analysis of phase change emulsion. J. Therm. Anal. Calorim..

[80] López-de-Dicastillo C., Gómez-Estaca J., Catalá R., Gavara R., Hernández-Muñoz P. (2012). Active antioxidant packaging films: Development and effect on lipid stability of brined sardines. Food Chem..

[81] Hsieh R.J., Kinsella J.E. (1989). Oxidation of polyunsaturated fatty acids: Mechanisms, products, and inhibition with emphasis on fish. Adv. Food Nutr. Res..

[82] Lorenzo J.M., Vargas F.C., Strozzi I., Pateiro M., Furtado M.M., Sant’Ana A.S., Rocchetti G., Barba F.J., Dominguez R., Lucini L. (2018). Influence of pitanga leaf extracts on lipid and protein oxidation of pork burger during shelf-life. Food Res. Int..

[83] Lobo V., Patil A., Phatak A., Chandra N. (2010). Free radicals, antioxidants and functional foods: Impact on human health. Pharmacogn. Rev..

[84] Gómez-Estaca J., López-de-Dicastillo C., Hernández-Muñoz P., Catalá R., Gavara R. (2014). Advances in antioxidant active food packaging. Trends Food Sci.Technol..

[85] Jayasena D.D., Jo C. (2013). Essential oils as potential antimicrobial agents in meat and meat products: A review. Trends Food Sci. Technol..

[86] Liu G., Xiong Y.L. (2000). Electrophoretic pattern, thermal denaturation, and in vitro digestibility of oxidized myosin. J. Agric. Food Chem..

[87] Sante-Lhoutellier V., Aubry L., Gatellier P. (2007). Effect of oxidation on in vitro digestibility of skeletal muscle myofibrillar proteins. J. Agric. Food Chem..

[88] Labuza T.P. (1968). Sorption phenomena in foods. Food Technol..

[89] Damodaran S., Parkin K.L. (2018). Química de Alimentos de Fennema.

[90] Stadtman E.R. (2006). Protein oxidation and aging. Free Radic. Res..

[91] Lund M.N., Luxford C., Skibsted L.H., Davies M.J. (2008). Oxidation of myosin by haem proteins generates myosin radicals and protein cross-links. Biochem. J..

[92] Abdou E.S., Galhoum G.F., Mohamed E.N. (2018). Curcumin loaded nanoemulsions/pectin coatings for refrigerated chicken fillets. Food Hydrocoll..

[93] Al-Hashimi A.G., Ammar A.B., Lakshmanan G., Cacciola F., Lakhssassi N. (2020). Development of a Millet Starch Edible Film Containing Clove Essential Oil. Foods.

[94] Sanchez-Gonzalez L., Chafer M., Chiralt A., Gonzalez-Martinez C. (2010). Physical properties of edible chitosan films containing bergamot essential oil and their inhibitory action on Penicillium italicum. Carbohydr. Polym..

[95] Winther J.R., Thorpe C. (2014). Quantification of thiols and disulfides. Biochim. Biophys. Acta (BBA).

[96] Ojagh S.M., Rezaei M., Razavi S.H., Hosseini S.M.H. (2010). Effect of chitosan coatings enriched with cinnamon oil on the quality of refrigerated rainbow trout. Food Chem..

[97] Serra A.T., Matias A.A., Nunes A.V.M., Leitão M.C., Brito D., Bronze R., Silva S., Pires A., Crespo M.T., San Romão M.V. (2008). In vitro evaluation of olive-and grape-based natural extracts as potential preservatives for food. Innov. Food Sci. Emerg. Technol..

[98] Puupponen-Pimiä R., Nohynek L., Meier C., Kähkönen M., Heinonen M., Hopia A., Oksman-Caldentey K.M. (2001). Antimicrobial properties of phenolic compounds from berries. J. Appl. Microbiol..

[99] Efenberger-Szmechtyk M., Nowak A., Czyzowska A. (2021). Plant extracts rich in polyphenols: Antibacterial agents and natural preservatives for meat and meat products. Crit. Rev. Food Sci. Nutr..

